# Practical prescribing in cancer cachexia

**DOI:** 10.1097/SPC.0000000000000814

**Published:** 2026-07-08

**Authors:** Dan Monnery, Joanne Collins, Joanne Droney

**Affiliations:** a Faculty of Health and Life Sciences, University of Liverpool, Liverpool, UK; b The Clatterbridge Cancer Centre NHS Foundation Trust, Liverpool, UK; c The Christie NHS Foundation Trust, Manchester, UK; d The Royal Marsden NHS Foundation Trust, London, UK

**Keywords:** cancer cachexia, nutrition, olanzapine, pharmacotherapy

## Abstract

**Purpose of review:**

Patients with cancer cachexia have shorter survival, decreased response to therapy, functional decline, and poorer quality of life. Optimal nutritional care consists of a multimodal approach, including screening, dietary counselling, nutritional supplementation, exercise advice, and consideration of prescribable medications. The aim of this article is to review the recent evidence supporting prescribing options in cancer malnutrition and cachexia.

**Recent findings:**

The use of olanzapine is supported by high-quality studies and meta-analyses and appears to lead to appetite stimulation, weight gain, and reduced grade 3 chemotherapy-related toxicities. The effect may be greater in those with nausea as a contributing factor to anorexia; and whilst the safety profile of olanzapine appears acceptable, some concerns about tolerability, leukopenia, and functional decline are noted.

The evidence for steroids and progestogens is outdated, and more recent studies have highlighted that these medications may worsen cachexia.

Low-level evidence exists for mirtazapine and N-3 fatty acids containing DHA.

**Summary:**

The role of pharmacotherapy in the management of cancer cachexia is limited, but when indicated, olanzapine has the strongest evidence to support its use and should displace steroids and progestogens as the first-line medication used in supportive oncology and palliative care.

## INTRODUCTION

Preserving and optimising nutritional health through cancer treatment is a significant challenge. Chemotherapy, radiotherapy, immunotherapy, and cancer surgery can all impair patients’ nutritional health [1] by reducing oral intake, leading to sarcopenia associated with chronic disease [2]. Poor nutritional health can, in turn, correlate with poorer outcomes, including a greater prevalence of treatment toxicities and worse survival from as little as 2.4% weight loss [1]. Worse nutrition also plays a role in poorer quality-of-life outcomes [3] and can alter the pharmacodynamics and pharmacokinetics of antineoplastic agents, such that dose-limiting treatment toxicities can limit the capacity for optimal anticancer treatment to be delivered [4] or can contribute to higher rates of treatment failure [5].

In addition to (but distinct from) malnutrition in cancer, in more advanced stages of the disease, cancer cachexia is a syndrome characterised by anorexia and extreme weight loss (of both adipose tissue and muscle) driven by systemic, disease-driven inflammation [2,6]. Cancer patients with cachexia have reduced survival, decreased response to therapy, functional decline, and poorer quality of life [7,8].

When nutritional optimisation is delivered alongside anticancer treatment, it can result in improved clinical outcomes, improved quality of life, and functional and emotional well-being [1]. However, given the various mechanisms contributing to malnutrition and cachexia in cancer, this often involves more than simply adding calories [6], and studies have demonstrated that increasing calorie intake either orally [9] or parenterally [10] does not, in isolation, reverse the poor outcomes associated with cancer cachexia.

**KEY POINTS**

- Cancer cachexia is a severe weight-loss syndrome characterized by anorexia, extreme weight loss, and muscle catabolism driven by systemic inflammation. Cancer patients with cachexia have shorter survival times, decreased responses to therapy, functional decline, and poorer quality of life.
- The optimal management of cachexia includes a multi-modal approach, including widespread screening, early nutritional intervention (e.g., dietary supplementation), exercise advice, and potentially pharmacological management of anorexia and associated symptoms.
- Olanzapine has the strongest evidence in the management of cancer anorexia among the available prescribable options in the UK and should displace corticosteroids, which are over-used in this setting given their low-level evidence. Olanzapine, however, may only offer short-term improvements in appetite.
- Further medications, based on the mechanism of cancer anorexia and cachexia, are in development and are likely to offer further promising alternatives for pharmacological management in the coming years.

The preferred approach to nutritional optimisation through cancer treatment, therefore, needs to be multimodal, commencing preferably before anticancer treatment begins and monitored regularly with clear thresholds for specialist input and nutritional support. Guidance on this multimodal approach has been published by the European Society for Clinical Nutrition and Metabolism (ESPEN) and includes screening, dietary counselling, nutritional supplementation, exercise advice, and consideration of prescribable medications [11]. Furthermore, the European Society for Medical Oncology (ESMO) guideline on cancer cachexia in adults also endorses this multimodal approach, whilst also considering the role of appetite stimulants, including corticosteroids, progestins, and olanzapine [12]. Whilst the American Society of Clinical Oncology (ASCO) guidelines do not recommend the routine use of appetite stimulants for cancer cachexia, they advise that clinicians may consider offering a short trial of medication aimed at reducing anorexia and improving body weight [8].

The aim of this article is to outline the prescribing options in cancer malnutrition and cachexia, and to describe recent evidence which may influence prescribing options for patients receiving cancer treatment.

## APPETITE STIMULANTS

### Olanzapine

Andrade *et al*. proposed that appetite stimulation and weight gain from the off-label use of atypical antipsychotics may be therapeutically exploited in cancer cachexia [13]. Olanzapine’s broad receptor profile, including antagonism at serotonergic, dopaminergic, and histaminergic receptors, underpins its potential to enhance appetite and reduce nausea [14].

Several studies have provided early evidence to support olanzapine for cancer cachexia [15,16]. However, the pivotal evidence for olanzapine comes from a 12-week, double-blind, placebo-controlled trial in which 124 patients with advanced lung, pancreaticobiliary, or stomach cancer receiving chemotherapy were randomised to low-dose olanzapine (2.5 mg daily) or placebo [17▪▪]. At least 5% weight gain occurred in 60% of patients in the olanzapine arm versus 9% of patients in the placebo arm (*P* < 0.001). Substantial appetite improvement was seen in 43% of the olanzapine arm versus 30% of patients receiving the placebo (*P* < 0.001). Improvements in cachexia-related quality of life and reductions in grade 3 chemotherapy-related toxicity were also observed [17▪▪].

Not all trials, however, show durable benefits. A recent double-blind, randomised, placebo-controlled trial investigated the effect of a short course of olanzapine 5 mg daily on cancer cachexia-associated anorexia in patients with no significant baseline nausea [18]. It demonstrated an early appetite improvement after one week, but this effect was not sustained by day 29 compared to placebo. Importantly, the study reported substantial attrition, raising concerns about selective retention, and interpretation is further limited by the short duration of follow-up and restriction to patients without significant baseline nausea. Olanzapine was associated with higher rates of grade ≥ 3 anaemia and leukopenia, as well as a significant decline in handgrip strength.

Furthermore, a recent prospective interventional study compared olanzapine 5 mg once daily with mirtazapine 15 mg nightly for four weeks in 152 patients with advanced oral cavity cancer and anorexia–cachexia [19]. Both improved appetite without meaningful weight gain. Whilst mirtazapine produced greater benefits in appetite, sleep, anxiety, and mood, olanzapine caused greater daytime somnolence and fatigue.

Despite these mixed trial findings, two recent meta-analyses support olanzapine’s role in cancer cachexia. Yuan *et al*. considered seventeen studies with 3457 participants and found that olanzapine significantly improved appetite and increased the proportion of patients achieving > 5% weight gain versus placebo and versus active controls, although mean weight change was not consistently significant [20^▪^]. Chen *et al*. analysed seven randomised controlled trials (RCTs) and reported one of the largest pooled weight gains (MD: 4.6 kg; 95% CI: 0.83–8.37) and the lowest odds of adverse events versus placebo (OR 0.26; 95% CI: 0.07–0.94), with sedation as the main side effect and milder metabolic changes at cachexia-treating doses [21^▪^].

Together, these studies indicate that olanzapine is a clinically promising option for cancer cachexia, although the effect may be greater in those whose appetite is affected by symptoms such as nausea and vomiting, which olanzapine is effective at managing. Strong trial data demonstrate improvements in appetite, weight, and quality of life, while pooled analyses suggest an acceptable safety profile and competitive efficacy relative to other pharmacologic agents. Findings from Othman *et al*., which highlighted haematological toxicity and functional decline, underscore the importance of larger, longer-duration trials to better define tumour-specific efficacy, optimal dosing strategies, and long-term safety monitoring [18].

### Corticosteroids

The use of steroids in the management of cancer-related anorexia relates to studies initially carried out nearly 50 years ago [22]. The most commonly used steroids for appetite stimulation are dexamethasone, prednisone, and methylprednisolone [12].

The predominant benefit of steroids in patients with cachexia is appetite stimulation; however, a systematic review in 2005 demonstrated appetite stimulation without weight gain [23]. The beneficial effects on appetite stimulation may be mediated through corticosteroids’ anti-inflammatory (glucocorticoid) activity, their influence on neuropeptide Y pathways involved in the regulation of food intake, and their additional anti-nausea properties [24]. Any weight gain associated with corticosteroids is more likely to result from increased fat deposition and fluid retention, which is related to their mineralocorticoid, sodium-retaining effects, rather than an increase in lean body mass [25].

The beneficial effects on appetite tend to wane after a few weeks, and more prolonged use may have harmful consequences, which exacerbate the clinical manifestations of cancer cachexia, including a reduction in muscle mass through the inhibition of protein synthesis [23,26^▪^].

### Progestogens

Progestogens are a class of steroid hormones (natural or synthetic) that act on progesterone receptors in the body. Medroxyprogesterone acetate and megestrol acetate have been evaluated for their roles in the management of cancer-related anorexia and weight loss.

Cochrane reviews (most recent 2018, updated from 2005 and 2013) found a low level of evidence for slight increases in weight with megestrol acetate (MA) (mean difference between MA and placebo: 1.45 kg, 95% CI: 0.15–2.75) [27]. Two more recent large systematic reviews and meta-analyses, however, conclude that megestrol acetate does not have a beneficial effect on appetite or weight gain [28,29]. The earlier systematic review of 23 clinical trials examining the use of megestrol acetate in cancer-related anorexia concluded (based on eight studies with weight as a study outcome) that high-dose megestrol acetate (> 320 mg/day) was associated with weight loss rather than weight gain [29]. A later meta-analysis of 13 studies involving 1,229 participants found a linear dose–response relationship with body weight, although the overall effect size was small [28]. Progestogens are associated with significant side effects, including thromboembolism, adrenal insufficiency, and hypogonadism [12].

### N-3 fatty acids

The European Society of Clinical Nutrition and Metabolism (ESPEN) recommends that, in patients receiving chemotherapy and at risk of weight loss or malnourishment, dietary supplementation with long-chain N-3 fatty acids or fish oil can improve appetite, lean body mass, and quality of life [11]. Eicosapentaenoic acid (EPA) (a long-chain omega-3 (n-3) polyunsaturated fatty acid derived from fish oil) has been shown to inhibit cancer-induced lipolysis and muscle protein breakdown by inhibiting IL-6 and proteolysis-inducing factor [30,31]. This anti-inflammatory mechanism may make it more suitable for cancer cachexia, in which pro-inflammatory mediators form part of the pathogenesis. A recent meta-analysis of the role of N-3 fatty acids in advanced non-small cell lung cancer described five studies (354 patients), in which N-3 fatty acids demonstrated a positive impact on weight gain (mean difference 1.22 kg, 95% CI: 1.05–1.38) compared to controls, but not on increased skeletal mass [31]. Furthermore, there was a greater improvement in health-related quality of life and physical functioning in patients taking N-3 fatty acid supplementation compared to controls (Global Health [MD: 14.40, 95% CI: 9.22–19.59]) [31]. The authors of this study commented that their findings stood in contrast to previous studies, which had shown no difference from dietary supplementation with eicosapentaenoic acid (EPA) [32]. They theorised that the reason for this was that this meta-analysis included studies in which docosahexaenoic acid (DHA) was included within the N-3 fatty acid supplementation, and that DHA, either alone or in combination with EPA, may prove more effective in improving weight and health-related quality of life [31].

Furthermore, a recent randomised controlled trial in patients receiving active treatment for breast cancer demonstrated that patients receiving EPA/DHA supplementation *and* vitamin D supplementation (50,000 units weekly) achieved greater weight gain (0.7 kg [± 0.4]), improved dietary intake, and higher plasma albumin compared to controls, but also to a greater extent than patients receiving either EPA/DHA supplementation *or* vitamin D supplementation alone [33^▪^].

These publications provide early evidence that dietary supplementation with N-3 fatty acids may be effective in combating weight loss and cachexia; however, there may be benefit from ensuring that the N-3 fatty acid supplementation contains DHA as well as EPA, and there may be additional benefit to be had from co-supplementing with vitamin D.

### Mirtazapine

Mirtazapine is an antidepressant that has been studied for its potential impact on increasing nutritional intake through appetite stimulation [34] and has historically been used off-licence as an appetite stimulant within palliative care. In a recent study, Arrieta *et al*. demonstrated that, compared to placebo, mirtazapine given at a dose of up to 30 mg/24 h to patients with advanced non-small cell lung cancer did not result in an improvement in self-reported appetite scores. However, patients receiving mirtazapine were noted to have a higher energy intake at 4 weeks (379.3 kcal; 95% CI: 1382.6–576.1), mainly from increased fat intake. This increase in energy intake compared with those in the placebo arm was maintained at 8 weeks, although there was no difference in weight change between groups [34]. A further recent randomised controlled trial by Chowdhury *et al*. examined the impact of mirtazapine 15 mg per day compared to 160 mg daily of megestrol acetate [35]. This study included an advanced mixed-cancer population and demonstrated that both mirtazapine and megestrol acetate improved appetite and body weight at 4–8 weeks compared to baseline, but there were no statistically significant between-group differences observed [35].

These studies, therefore, indicate that whilst there may be some positive benefit from mirtazapine in terms of appetite stimulation and energy consumption, the findings are mixed, and it does not appear superior to other tested medications.

### Novel agents

As the mechanisms underlying cancer cachexia are studied, novel targeted agents are increasingly being proposed. Growth differentiation factor 15 (GDF-15) has emerged as a key component of anorexia and body-weight regulation and is implicated in the pathogenesis of cachexia [36]. Groarke *et al*. proposed ponsegromab, a monoclonal antibody targeting GDF-15 as a therapeutic agent to treat cancer cachexia [37]. In their recently published phase 2 randomised controlled trial, ponsegromab, compared to placebo, demonstrably improved weight gain in patients with advanced cancer in a dose-dependent manner over a 12 week study period, with patients in the highest dose category gaining a clinically important > 5% body weight [37]. Furthermore, patient-reported symptoms recorded using the Functional Assessment of Anorexia Cachexia Treatment–Anorexia Cachexia Subscale (FAACT-ACS) were also superior to placebo, although this difference was not dose-dependent [37]. Whilst not currently commercially available pending further trials, ponsegromab appears to be a promising future intervention for cancer cachexia.

A further potential future intervention, anamorelin, a ghrelin receptor agonist, has previously been analysed in two meta-analyses and shown to improve body weight, lean body mass, and patient-reported quality of life [38,39]. However, anamorelin has not received approval from any national drug authorisation committees and, therefore, whilst it is cited as having intermediate evidence of moderate benefit within ASCO guidelines [8], it is not currently commercially available.

## CONCLUSION

Current evidence indicates that pharmacotherapy offers only a modest contribution to the multimodal management of cancer-related cachexia. Within supportive oncology and palliative care, it remains essential to recognise the substantial and sometimes serious harms associated with traditional interventions such as corticosteroids. A more judicious approach requires proactive, coordinated engagement with the multidisciplinary team to ensure timely identification, comprehensive assessment, and holistic management of this complex and multifactorial syndrome.

Traditionally, whilst corticosteroids have been the mainstay of pharmacological intervention for anorexia in palliative care, the evidence underpinning this is old and is being superseded by modern trials of alternative medications. Based on the results of the study by Sandaya *et al*., along with supporting evidence from other studies [17▪▪,^40^], a rapid recommendation update of the ASCO guideline for the management of cancer cachexia was published in 2023, stating that low-dose olanzapine (2.5–5 mg, preferably at bedtime) can be offered to improve appetite and weight gain in advanced cancer patients, whilst moving both corticosteroids and progesterone analogues to second-line options, to be used only when a patient cannot tolerate low-dose olanzapine [41].

However, within palliative care, prevailing prescribing practices for corticosteroids have been slow to change. This may be due to the ease of prescribing corticosteroids and the wide-ranging familiarity with these medications as a therapeutic option, and it may also reflect the overall sense of well-being reported by patients anecdotally when given steroids. However, in the shifting landscape of delivering supportive oncology to patients alongside anticancer treatment for a longer duration, the role of steroids in the management of cachexia is limited.

Low-dose olanzapine is a simple, inexpensive, and generally well-tolerated option for cancer cachexia. While full guideline endorsement continues to develop, the growing evidence base strongly supports its value as a practical, multimodal agent, which may improve appetite while simultaneously easing nausea, anxiety, and sleep disturbances shortly after initiation. We therefore believe that prescribing practices within supportive oncology and palliative care should shift in favour of using olanzapine as a first-line treatment. Whilst other medications show mixed efficacy with some low-level indication of therapeutic remit, these should be secondary options and based on an individual assessment of risks and benefits. Table 1 outlines the hierarchy of appetite stimulants that can be prescribed in the UK based on the outcome of this review.

**Table 1.** Appetite stimulants used in the UK (unlicensed indication

Drug/Class	Formulation/route	Typical starting dose	Common dose range	Main use	Important notes/risks
Olanzapine	Oral tablets; orodispersible tablets (ODT)*	2.5 mg nocte	2.5–5 mg nocte (occasionally up to 10 mg)	Appetite stimulation, nausea, weight gain, insomnia/anxiety	Sedation common; may help most when nausea coexists. Monitor for delirium, orthostasis, metabolic effects. ASCO 2023 conditionally supports low-dose olanzapine as an option for cancer-related anorexia/cachexia based on emerging RCT evidence. [17^▪▪^] Usual course length is up to 12 weeks.
Dexamethasone	Oral tablets/solution; IV; IM; SC (off-label palliative use)	2–4 mg mane	2–8 mg/day (short-term)	Rapid appetite/energy improvement (short-term use)	Class effect evidence in cachexia. Often works quickly but benefit wanes after weeks. Hyperglycaemia, myopathy, insomnia, infection risk, delirium. Best for short prognosis or acute symptom relief. Trial for ~5–7 days often used
Prednisolone	Oral tablets; oral soluble tablets/liquid	5–10 mg mane	10–20 mg mane (short-term)	Appetite stimulation (short-term use)	Similar steroid cautions; less potent than dexamethasone. No cachexia-specific dosing trials. Dose extrapolated from corticosteroid class effect and clinical equivalence with dexamethasone. If long-term use of a corticosteroid is contemplated, a switch to the non-fluorinated **prednisolone** 10–20 mg/day PO should be considered. However, for patients expected to live months rather than weeks, progestogens may be more appropriate. [8]
Megestrol acetate	Oral tablets; oral suspension	160 mg daily	160–800 mg/day (in two or more divided doses)	Appetite and weight gain	Weight gain mostly fat/fluid rather than lean muscle. Risk of VTE, oedema, adrenal suppression. Avoid in high thrombosis risk where possible. No clear additional benefit above moderate doses [8,12,42]. Treatment should be continued for at least 6 weeks
Medroxyprogesterone acetate	Oral tablets	400 mg daily	400–1000 mg daily	Appetite stimulation	Similar concerns to megestrol; less commonly used in some centres [43]
Mirtazapine	Oral tablets; orodispersible tablets (ODT) → oral dissolution	7.5–15 mg nocte	15–30 mg nightly	Appetite + depression/anxiety/insomnia	Trial-based dosing only. Helpful if mood disorder or insomnia coexist. Evidence for cachexia itself is weaker than for olanzapine/megestrol. RCTs show inconsistent benefit for appetite/weight gain in cancer cachexia.
					Use is extrapolated from antidepressant dosing. Sedation common at low dose [44,45]

*Olanzapine ODT may be administered sublingually, note that this route is unlicensed.
